# Vascular recanalization exacerbates BBB permeability after ischemic stroke

**DOI:** 10.3389/fneur.2025.1682748

**Published:** 2025-10-01

**Authors:** Rong-Fei Wang, Jing Liu, Chang-Hui Chen

**Affiliations:** ^1^Guangzhou University of Chinese Medicine, Guangzhou, China; ^2^Department of Neurology, The Third Affiliated Hospital of Guangzhou University of Chinese Medicine, Guangzhou, China; ^3^Department of Neurology, The Affiliated Brain Hospital of Guangzhou Medical University, Guangzhou, China

**Keywords:** ischemic stroke, recanalization, blood–brain barrier, tight junctions, transcytosis

## Abstract

**Introduction:**

Ischemic stroke is a common and serious neurological disease. After cerebral ischemia occurs, the integrity of the BBB is disrupted, leading to increased permeability, causing pathophysiological changes such as brain edema and hemorrhagic transformation, which aggravates neuronal damage. Such changes become more obvious after the recovery of blood flow. However, the effect of vascular recanalization on blood–brain barrier leakage is poorly known.

**Methods:**

Mice were divided into the recanalization group and the non-recanalization group. Mice in the recanalization group suffered from the middle cerebral artery occlusion and were reperfused 60 min later. Mice in the non-recanalization group suffered from permanent occlusion of the middle cerebral artery. The permeability of the blood–brain barrier was tested using fluorescence staining, and the expression of tight junction proteins and transcytosis-related proteins were analyzed by western blot.

**Results:**

The IgG results revealed a significantly larger area of leakage in the recanalization group compared to the non-recanalization group. A consistent trend was observed in the FITC-dextran leakage experiment. Moreover, after blood flow recanalization, there was a significant reduction in tight junctions-related proteins, occludin and ZO-1. Meanwhile, both ischemia and reperfusion caused changes in the ratio of transcytosis related protein Caveolin-1 /MFSD2a, and this is more obvious in the blood flow recanalization group.

**Conclusion:**

Vascular recanalization can exacerbate blood–brain barrier disruption, concurrently impairing both the paracellular and transcytosis pathways. This finding provides a rationale for exploring new approaches for protecting the integrity of the blood–brain barrier, reducing its permeability, and lowering the risk of hemorrhagic transformation.

## Introduction

Ischemic stroke is a serious neurological disease (1), characterized by high incidence, disability and mortality, posing a significant societal and economic burden. In the pathological process of cerebral ischemia, the blood–brain barrier (BBB) plays a crucial role, which is a special structure existing between blood and brain tissue. It prevents harmful substances from entering the brain, maintains the stability of the internal environment of the brain tissue, and ensures the normal function of neurons (2, 3).

Vascular recanalization represents a cornerstone therapeutic strategy for cerebral ischemia. Common clinical interventions such as intravenous thrombolysis and mechanical thrombectomy aim to reopen occluded blood vessels, restore blood supply to ischemic brain tissue as promptly as possible, and reduce neuronal death (4, 5). However, a growing number of studies indicate that while vascular recanalization restores blood flow, it may also lead to ischemia–reperfusion injury, which can further damage the BBB, potentially leading to hemorrhagic transformation in severe cases. Hemorrhagic transformation is a serious complication in the treatment of cerebral ischemia, and its occurrence is intimately linked to the disruption of the BBB. After cerebral ischemia occurs, particularly following recanalization therapy, the integrity of the BBB is compromised, and its permeability increases. This allows blood components such as red blood cells and plasma proteins to leak into the brain’s interstitial spaces, thereby triggering hemorrhagic transformation. Hemorrhagic transformation significantly increases patient disability and mortality rates (6). Research on BBB changes following vascular recanalization will help to explore new approaches for protecting BBB integrity, reducing its permeability, and lowering the risk of hemorrhagic transformation.

The BBB is a complex dynamic functional system composed of various cells and molecules. It is primarily composed of brain microvascular endothelial cells (BMECs), basement membrane, pericytes and astrocyte endfeet. These components work together to maintain the function of the BBB (7–9). BMECs are the main structural and functional basis of the BBB. They are interconnected to form a tight monolayer structure, which restricts the free passage of most substances. Alterations in BBB permeability involve multiple mechanisms, with changes in tight junction proteins and transcytosis play key roles.

Tight junction proteins are pivotal in maintaining the integrity of the paracellular pathway at the BBB (10, 11). Following cerebral ischemia, the expression and cellular distribution of key tight junction proteins such as claudin-5, occludin, and ZO-1 are markedly altered. These changes lead to the disruption of tight junction structure and an increase in BBB permeability (12, 13). During ischemia–reperfusion, for instance, levels of these proteins are significantly downregulated, and their localization shifts from a continuous linear pattern to fragmented or punctate arrangements along the cell membrane. This breakdown enlarges intercellular gaps and facilitates the paracellular leakage of substances into the brain tissue (14). Furthermore, activation of specific signaling pathways, such as the MAPK and NF-κB pathways, modulates the phosphorylation status of tight junction proteins and their interactions, thereby influencing tight junction stability and barrier function (15).

Under physiological conditions, transcytosis across BBB endothelial cells is limited, helping preserve barrier selectivity. However, cerebral ischemia, particularly upon reperfusion, leads to a pronounced upregulation of transcellular transport mechanisms. Caveolae-mediated transcytosis, in particular, has been implicated in the increased BBB permeability observed after ischemic events (16). This facilitates the vesicular transport of macromolecules such as albumin and immunoglobulins, compromising barrier integrity. Other mechanisms also contribute, for example, MFSD2a, a transporter known to suppress transcytosis by modulating lipid membrane organization, may also be involved in post-ischemic BBB dysfunction (17).

Given the critical involvement of both paracellular and transcellular pathways in ischemia–reperfusion-induced BBB disruption, there is a compelling need to investigate how reperfusion affects the dynamics of tight junction expression and reorganization, as well as the regulation of transcytosis activity. A deeper understanding of these processes could reveal novel therapeutic targets for preserving BBB integrity and mitigating neuronal damage following recanalization therapy.

## Materials and methods

### Animals and MCAO surgery

The experiments in this study were carried out on 8-9-week-old male C57BL/6 J mice (Guangdong Zhiyuan Biomedical Technology Co., LTD.). Before surgery, the mice were adapted in the home cage for 3–5 days, with a suitable illumination period and fixed room temperature, as well as free access to food and water. Modeling began when the weight of the mice reached 25 ± 3 g. All operations in this experiment were carried out under deep anesthesia to minimize pain. All experimental operations were carried out in accordance with the ethical guidelines of animal experiments of Guangzhou Medical University. Mice were randomly divided into two groups: the recanalization group and the non-recanalization group. The non-recanalization mice underwent a distal middle cerebral artery occlusion surgery (dMCAO) and the recanalization group underwent transient middle cerebral artery surgery (tMCAO). For the dMCAO surgery, mice were deeply anesthetized with isoflurane, and then a “U”-shaped incision was made in the left temporo-occipital region, and the parotid gland and surrounding soft tissues were retracted downward. The left middle cerebral artery was exposed and occluded with bipolar electric coagulation mice. For the tMCAO surgery, after the mice were fully anesthetized, skin was cut along the midline of the neck to expose the common carotid artery (CCA), external carotid artery (ECA), and internal carotid artery (ICA). Then, a 6–0 suture (Doccol, Massachusetts, USA) was inserted from the external carotid artery (ECA) into the internal carotid artery (ICA) to occlude the origin of the middle cerebral artery (MCA). Reperfusion was performed by withdrawn the suture 60 min after the occlusion.

### Tissue processing

One or three days after MCAO surgery, 0.05% fluorescein isothiocyanate (FITC-dextran, weighted 70 KD, invitrogene) solution was injected via the tail vein of the mice. One hour later, the mice were anesthetized with sodium pentobarbital (40 mg/kg, i.p.) and perfused transcardially with 0.9% saline followed by 4% paraformaldehyde (PFA) in chilled 0.1 M of phosphate buffer (PB). The brains were removed and fixed in a 4% PFA for 1 day, and then transferred to a 30% sucrose solution for dehydration. After that, the brains were cut into coronal sections with a thickness of 40 μm using a freezing microtome, and stored in cryopreservation solution at 4 °C.

### Immunofluorescence staining

Sections were rinsed with PBS and then incubated in 0.3% Triton X-100 solution for 30 min and subsequently 5% donkey serum for 60 min. Next, the sections were incubated in the primary antibody overnight at 4 ° C. The antibodies used were as follows: anti-rabbit ZO-1 (1:200, thermo fisher, 14-9776-80, 2083505), anti-rabbit occludin (1:100, Abcam, Ab216327, GR-3243495-3), anti-rat CD31 (1:100, BD, 550274,1207300), anti-rabbit Caveolin-1 (1:200, 3238s, CST, 3), anti-rabbit MFSD2a (1:100, ER-1912-98, Cambridge biosciencce, ER-1912-98). On the following day, sections were rinsed with PBS and incubated with the fluorescently labeled secondary antibody corresponding to the species of the primary antibody for 60 min. All of the fluorescently labeled secondary antibodies (Donkey anti-rabbit 488, 1:1,000 and Donkey anti-rat 594, 1:1,000) were ordered from thermo fisher. For the IgG staining, all procedures were the same to the immunostaining without primary antibody incubation, the anti-mouse 594 was incubated at room temperature for 2 h. Finally, the sections were mounted with an anti-fluorescence decay mounting agent containing DAPI, and coverslipped. Images were visualized under a laser confocal fluorescence microscope (ZEISS 900). Quantification was carried out using ImageJ software.

### Western-blot

The cortical region, including ischemic core was selected for protein extraction. Tissues were homogenized by ultrasonication on ice and then centrifuged. The protein concentrations were quantified. Equal amounts of protein were loaded on SDS-PAGE gels and transferred to PVDF membranes. For immunoblotting, the membrane was first blocked with 5% nonfat milk and then incubated with primary antibody at 4 °C overnight. The primary antibodies used were as follows: anti-ZO-1 (1:1,000), anti-Caveolin-1 (1:1,000), anti-occludin (1:1,000), anti-MFSD2a (1:1,000) and anti-GAPDH (1:2,000,). Semiquantitative analysis of the chemiluminescence signal was performed, and the gray values of all bands were standardized to that of the GAPDH band. Immunoblot band intensities were quantified using ImageJ software.

### Statistical analysis

All data were statistically analyzed using GraphPad prism 10 software. All experimental data were presented as mean ± standard deviation (SD) and analyzed by normality. When the data met the normality and homogeneity of variance tests, the t-test was used for comparison between the two groups. *p*-value < 0.05 was considered statistically significant.

## Result

### Reperfusion increased permeability of the BBB

**Figure 1:**
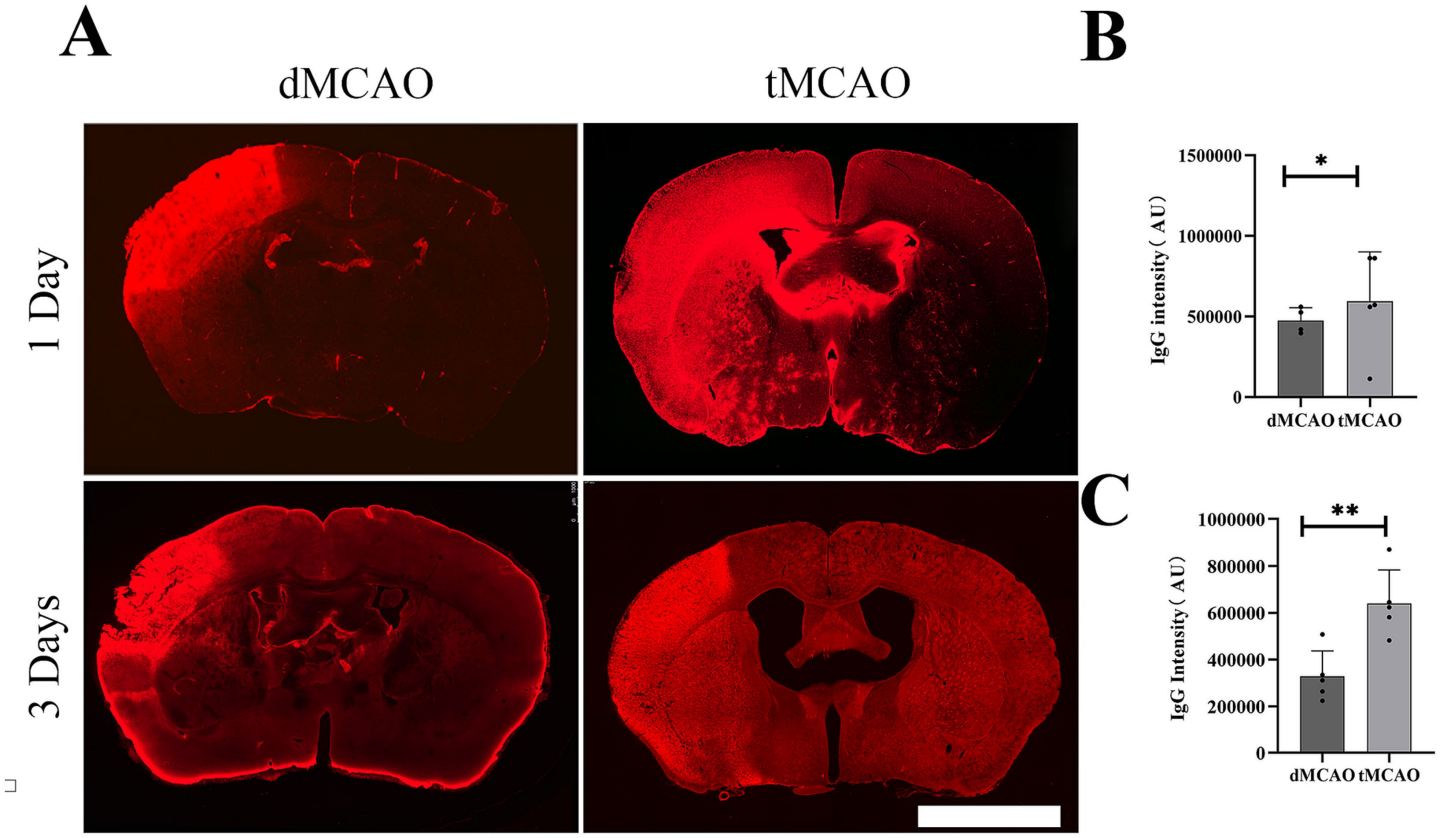
Comparison of IgG leakage. **(A)** Typical images showed IgG staining in the dMCAO group and the tMCAO group 1 day and 3 days after surgery. **(B)** The bar graph showed the quantification of IgG intensity in the cortex 1 day after MCAO. **(C)** The bar graph showed the quantification of IgG intensity in the cortex 3 day after MCAO. ^*^Indicates *p* < 0.05, ^**^indicates *p* < 0.01, *n* = 4–5 per group. Bar = 1 mm.

**Figure 2:**
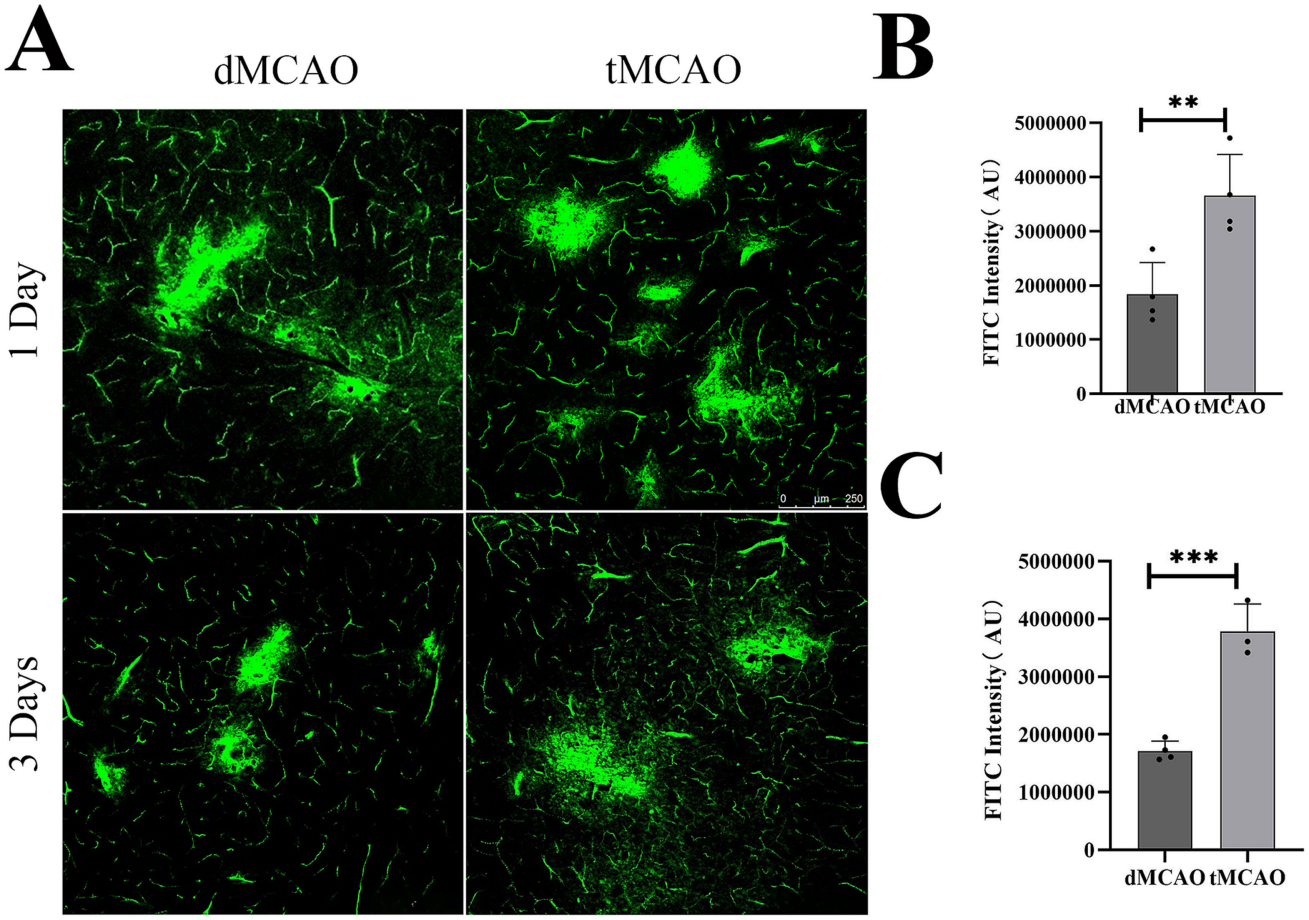
Comparison of FITC-detran extravasation. **(A)**Typical images showed FITC-detran leakage out of the vessel in the dMCAO group and the tMCAO group 1 day and 3 days after surgery. **(B)** The bar graph showed the quantification of FITC leakage intensity in the cortex 1 day after MCAO. **(C)** The bar graph showed the quantification of FITC leakage intensity 3 days after MCAO. ^*^Indicates *p* < 0.05, ^**^indicates *p* < 0.01, *n* = 3–4 per group. Bar = 250 um.

To evaluate the permeability of BBB, IgG immunohistochemistry was performed. The results revealed a significant increase in IgG leakage in the tMCAO group compared to the dMCAO group. An expanded IgG intensity was observed at 1 day after ischemia–reperfusion injury, which was significantly greater than that in the dMCAO group (Figure 1). This increase in permeability persisted for up to 3 days following cerebral ischemia. To further assess BBB integrity, FITC-dextran extravasation was quantified. The tMCAO group exhibited a marked elevation in FITC-dextran fluorescence intensity outside the vasculature compared to the dMCAO group (Figure 2). Together, these findings demonstrate that cerebral ischemia–reperfusion injury induces a significant and sustained increase in BBB permeability, which gradually recovers over time.

### Tight junction protein degradation in stroke mice

**Figure 3:**
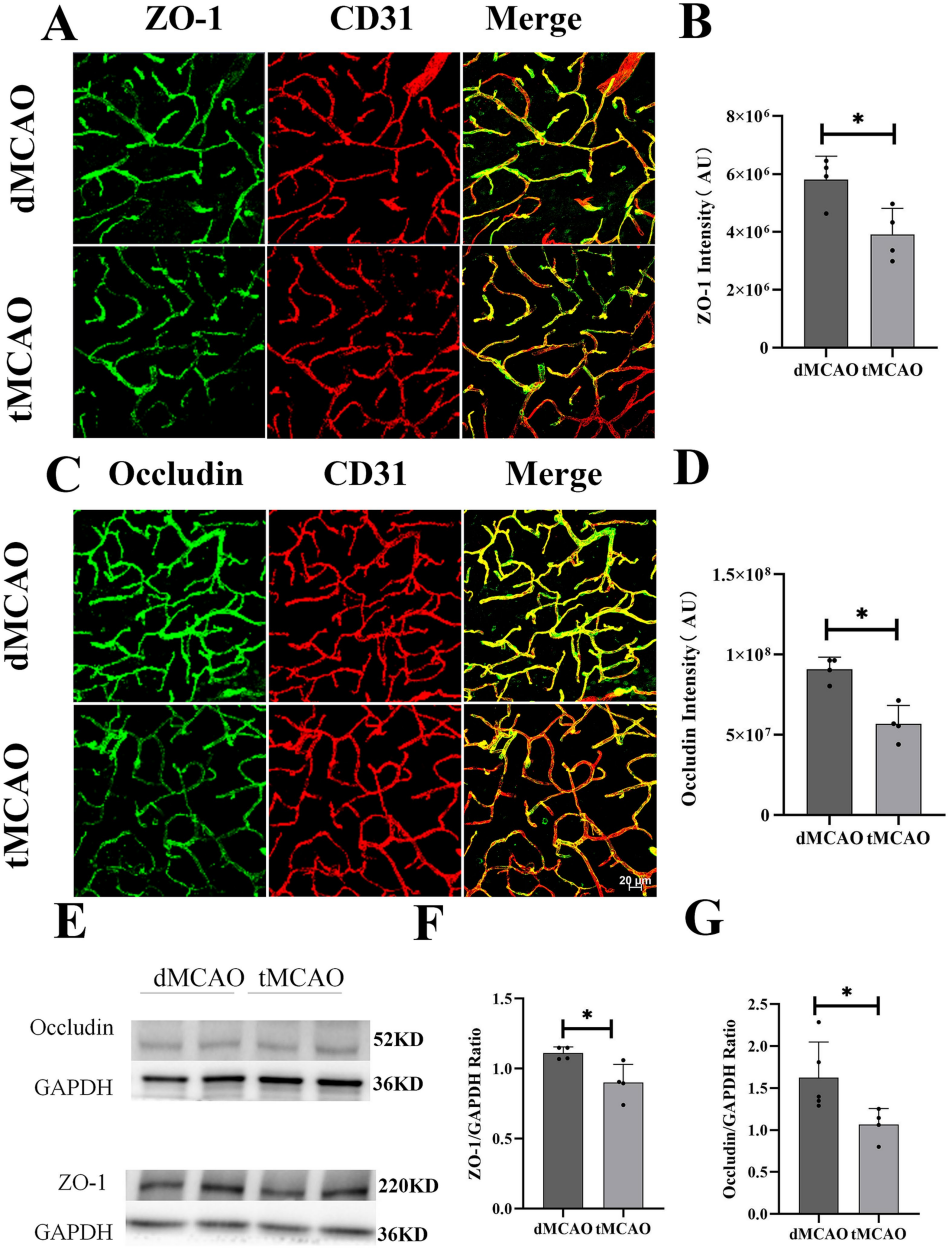
Comparison of ZO-1 and occludin expression. **(A)** Typical images showed ZO-1 expression 3 days after surgery. **(B)** Bar graph showed the quantification of ZO-1 intensity from the infarct site. **(C)** Typical images showed occludin expression 3 days after surgery. **(D)** Bar graph showed the quantification of occludin intensity from the infarct site. **(E)** Images showed western blot results of ZO-1 and occludin from the infarct site in the cortex. **(F,G)** Bar graph showed the quantification of ZO-1 and occludin expression from western blot. ^*^Indicates *p* < 0.05, ^**^indicates *p* < 0.01, and *n* = 3–5 per group. Bar = 20 um.

To investigate the effects of ischemia–reperfusion on paracellular permeability, the expression and localization of tight junction proteins ZO-1 and occludin were assessed using immunohistochemistry and Western blot. Immunofluorescence results (Figure 3) revealed mild alterations in the dMCAO group, where ZO-1 and occludin exhibited sporadic punctate discontinuities amid largely preserved continuous linear staining. In contrast, the tMCAO group showed severe disruption of tight junction integrity at 1 day after reperfusion. Both ZO-1 (Figures 3A,B) and occludin (Figures 3C,D) displayed fragmented staining patterns with extensive discontinuity and loss of membranous organization. Semiquantitative analysis of fluorescence intensity further confirmed a significant reduction in residual ZO-1 and occludin protein levels in the tMCAO group compared to the dMCAO group. Western blot analysis consistently demonstrated that protein expression of occludin (Figures 3E,F) and ZO-1 (Figures 3E,G) was significantly lower in the tMCAO group at all time points examined (*p* < 0.05), with a progressive decline over time (*p* < 0.05), providing quantitative evidence for the ongoing tight junction degradation. Together, these findings indicate that ischemia–reperfusion injury markedly accelerates the degradation of tight junction proteins and disrupts their structural organization, contributing to increased paracellular leakage.

### The enhanced transcytosis of the BBB in ischemia–reperfusion mice

**Figure 4:**
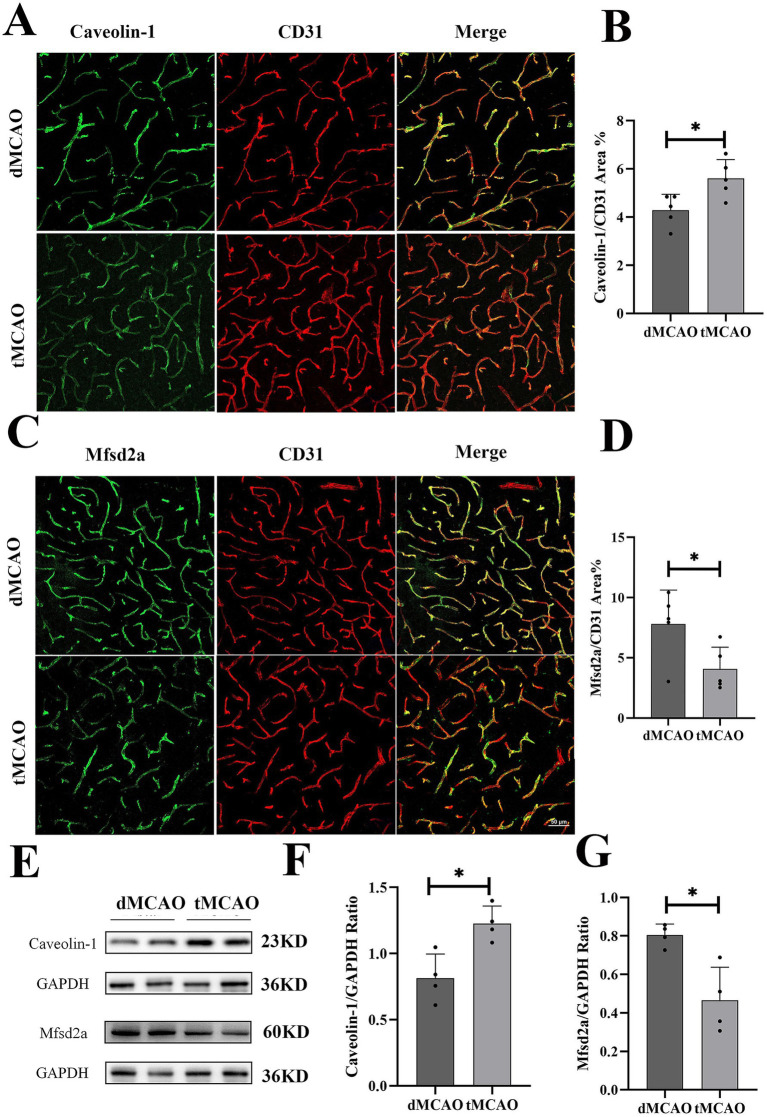
Comparison of Cavoelin-1 and MFSD2a expression. **(A)** Typical images showed Cavoelin-1 expression 3 days after surgery. **(B)** Bar graph showed the quantification of Cavoelin-1 covered CD31 positive area from the infarct site. **(C)** Typical images showed MFSD2a expression 3 days after surgery. **(D)** Bar graph showed the quantification of MFSD2a covered CD31 positive area from the infarct site. **(E)** Images showed western blot results of Cavoelin-1 and MFSD2a from the infarct site in the cortex. **(F,G)** Bar graph showed the quantification of Cavoelin-1 and MFSD2a expression from western blot. ^*^Indicates *p* < 0.05, ^**^indicates *p* < 0.01, and *n* = 3–5 per group. Bar = 50 um.

Previous studies have demonstrated that caveolae-mediated endocytosis represents the principal transcytotic pathway at the BBB (7). Following acute ischemic stroke, transcytosis has been reported to dominate endothelial transcellular transport in the central nervous system (18–20). Key regulators of caveolae-mediated transcytosis include caveolins (Cav) and MFSD2a (16, 21). To evaluate the impact of recanalization on transcytotic pathway, we examined the expression and distribution of Caveolin-1 and MFSD2a, critical determinants of caveolar transcytosis activity. In the dMCAO group, Caveolin-1 expression was moderately upregulated but retained a relatively uniform distribution. In contrast, the tMCAO group exhibited a substantial increase in Caveolin-1 expression, with pronounced aggregation of positive staining in brain microvascular endothelial cells 1 day after reperfusion (Figures 4A,B). Western blot analysis further corroborated these findings, demonstrating significantly elevated Caveolin-1 levels in the tMCAO group (Figures 4E,F). Concurrently, MFSD2a protein expression was significantly reduced in the tMCAO group at all time points (Figures 4C,D) and exhibited a progressive decline over time, as confirmed by Western blot (Figures 4E,G). Collectively, these results indicate that ischemia–reperfusion injury enhances caveolae-mediated transcytosis in vascular endothelial cells.

## Discussion

Vascular recanalization leads to a significant increase in BBB permeability through multiple interconnected mechanisms, primarily involving inflammation, oxidative stress, and hemodynamic changes. Upon reperfusion, infiltrating inflammatory cells such as neutrophils and monocytes release cytokines including TNF-*α*, IL-1β, and IL-6. These promote BBB disruption by upregulating adhesion molecules like ICAM-1, activating NF-κB signaling, and directly degrading tight junction proteins, thereby facilitating IgG extravasation (22–24). Concurrently, ischemia–reperfusion induces oxidative stress due to mitochondrial dysfunction and excessive reactive oxygen species (ROS) production, which oxidizes tight junction proteins and activates matrix metalloproteinases (MMPs), further compromising BBB integrity (25–27). Additionally, restored blood flow imposes mechanical stress on endothelial cells, triggering cytoskeletal reorganization and MAPK pathway activation, which alters tight junction protein phosphorylation and reduces barrier stability (28). Hemodynamic changes may also amplify inflammation and ROS production, collectively exacerbating IgG leakage. In our study, we observed that the permeability of the BBB can be influenced in two distinct ways, regardless of whether vessels are recanalized or not. Importantly, both pathways lead to a significant increase in leakage after vascular recanalization. This finding holds considerable clinical relevance, suggesting that strategies to prevent pathological changes resulting from BBB disruption may need to be tailored depending on the recanalization status. For instance, disruption of tight junctions may allow massive infiltration of inflammatory cells, blood-derived substances, and even erythrocytes into the brain parenchyma—potentially a key mechanism underlying recanalization-induced hemorrhagic transformation. In contrast, receptor-mediated transcytosis primarily facilitates selective transport of specific molecules. Its upregulation after recanalization may contribute to distinct pathological changes within the brain tissue.

The degradation of tight junction proteins is a central event in BBB disruption following cerebral ischemia, driven by interconnected inflammatory, oxidative, and signaling pathways. Inflammatory factors such as TNF-*α* and IL-1β, released post-ischemia, activate the NF-κB pathway, leading to upregulation of MMPs—particularly MMP-2 and MMP-9—which specifically degrade key tight junction proteins like Claudin-5 and occludin. Inhibition of TNF-α has been shown to attenuate this degradation and reduce BBB leakage (15). Concurrently, oxidative stress contributes significantly through the excessive generation of ROS and reactive nitrogen species (RNS), which directly oxidize tight junction components, disrupting their stability and interactions. Furthermore, ROS indirectly promote tight junction degradation by activating MMPs (29). Intracellular signaling pathways, including MAPK (ERK, JNK, p38) and PI3K/Akt, are aberrantly activated in ischemia and enhance tight junction disassembly via phosphorylation of proteins such as occludin, altering their localization and function. Recently, the ubiquitin-proteasome system has been implicated in tight junction regulation, with studies showing that USP14 targets ZO-1 for degradation. Inhibition of USP14 alleviates ischemia-induced BBB damage, suggesting a novel therapeutic target, though its role in hemorrhagic transformation remains unclear (30). In our study, we observed that compared to non-recanalized mice, the degradation of tight junction proteins was significantly more pronounced under recanalization conditions. This indicates that vascular recanalization alone may be insufficient to ensure a favorable prognosis. Successful recanalization is only the first step, and subsequent efforts must focus on protecting the BBB. Our finding provides an important theoretical basis for developing combination therapies, optimizing treatment timing, and achieving personalized treatment.

Vascular recanalization after cerebral ischemia enhances transcytosis through synergistic activation of multiple signaling pathways. The PI3K/Akt pathway is significantly activated during ischemia–reperfusion, promoting the expression and membrane translocation of the Caveolin-1/MFSD2a axis, which increases caveolae formation and transcytosis activity (31). Concurrently, the MAPK pathway (including ERK, JNK, and p38 MAPK) is activated by oxidative and ischemic stress, leading to phosphorylation of transcription factors that upregulate Caveolin-1/MFSD2a expression and further potentiate transcytosis (32). Additionally, hypoxia-induced VEGF release from astrocytes and neurons binds to endothelial receptors, inducing cytoskeletal rearrangement that facilitates vesicle transport and upregulating caveolin-mediated transcytosis proteins (33). Thus, the PI3K/Akt, MAPK, and VEGF pathways collectively enhance transendothelial vesicular trafficking following recanalization (34). Interestingly, our results indicate that vascular recanalization can also lead to transcytosis-mediated vascular leakage. This indicates that recanalization disrupts the BBB through a “dual-hit” mechanism. Therapeutic strategies targeting only the restoration of tight junctions (e.g., by inhibiting MMPs) may be insufficient to fully preserve BBB integrity, and thus must also consider suppressing abnormal transcytosis. This provides a more comprehensive theoretical basis for combination therapy. The activation of transcytosis may explain why, even in the absence of severe hemorrhagic transformation, specific harmful macromolecules—such as fibrinogen, IgG, and inflammatory factors—accumulate in the brain after recanalization, thereby driving specific neuroinflammatory responses and toxic effects. This offers a new perspective for understanding the complex pathological changes following recanalization.

There are limitations in the current study. First, our assessment of blood–brain barrier (BBB) integrity relied on the analysis of zo-1 and occludin expression. Although these are key components of tight junctions, we did not investigate claudin-5, which is the primary transmembrane protein critical for paracellular seal formation. The absence of claudin-5 data limits a comprehensive mechanistic understanding of the observed changes in BBB permeability. Future studies employing validated antibodies and techniques are necessary to specifically define the role of claudin-5 in this context. Second, this study was conducted exclusively in male animal models. Consequently, our findings cannot be generalized to females, as sex is a crucial biological variable that can profoundly influence neurovascular pathophysiology and inflammatory responses. The lack of female data precludes any analysis of potential sex-specific differences and represents a significant gap in the generalizability of our conclusions. Future work must include both sexes to determine the translatability of these mechanisms across populations.

## Conclusion

In summary, our study reveals that vascular recanalization concurrently disrupts both paracellular and transcellular transport pathways, thereby exacerbating BBB permeability. This finding underscores that successful recanalization is merely the initial step; comprehensive neuroprotection and improved patient outcomes ultimately require a multi-target therapeutic strategy that simultaneously reinforces tight junctions and suppresses pathological transcytosis.

## Data Availability

The raw data supporting the conclusions of this article will be made available by the authors, without undue reservation.
