# Supplementary figures and images for: Vascular recanalization exacerbates BBB permeability after ischemic stroke
Source: Front Neurol. 2025 Oct 1;16:1682748. doi: 10.3389/fneur.2025.1682748 (PMC12520948; doi:10.3389/fneur.2025.1682748)

**Occludin**

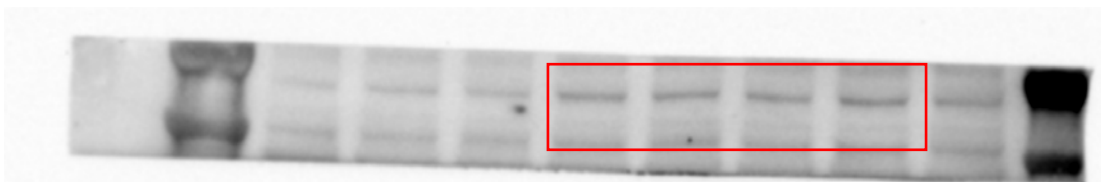

**GAPDH**

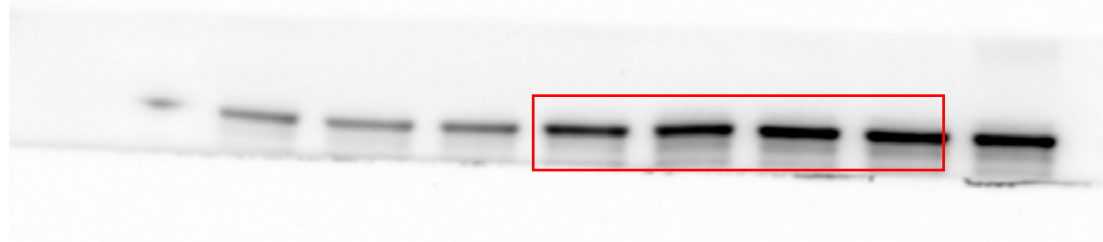

**ZO-1**

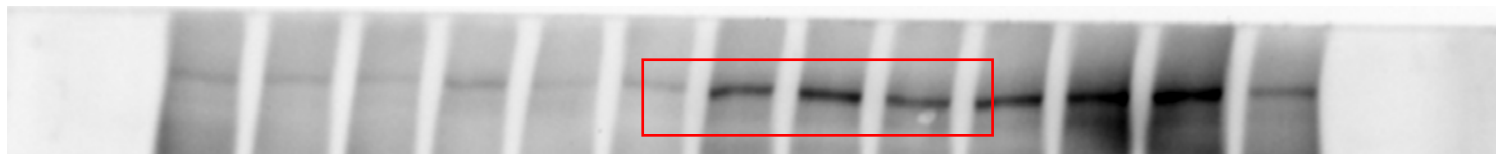

**GAPDH**

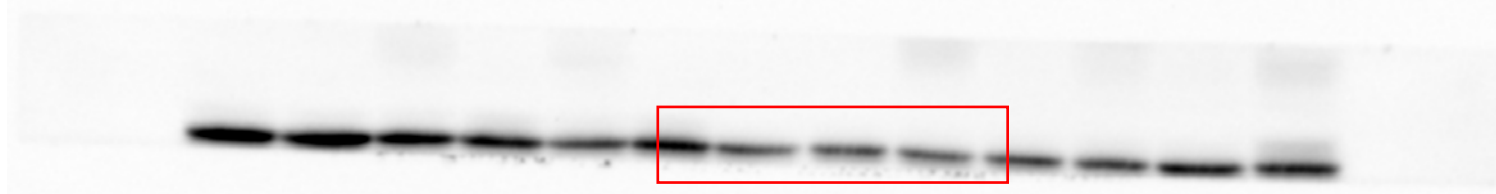

**Caveolin-1**

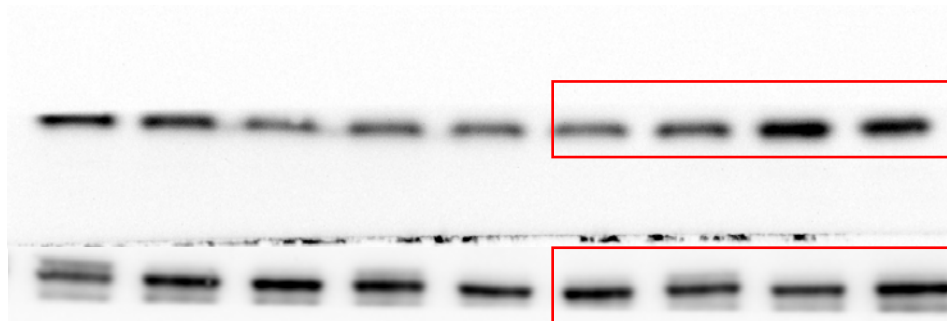

**GAPDH**

**MFSD2a**

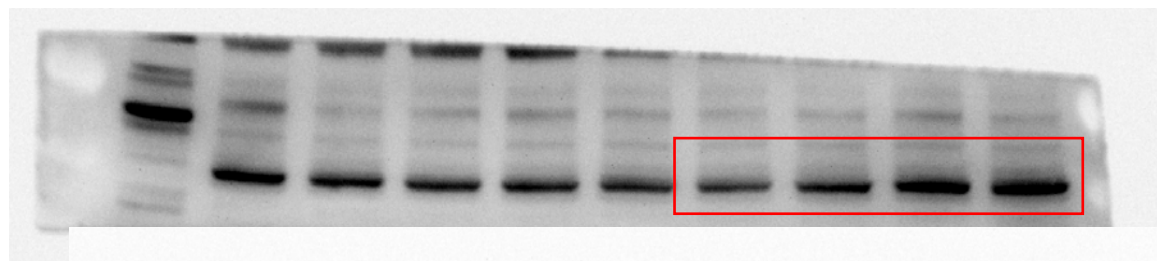

**GAPDH**

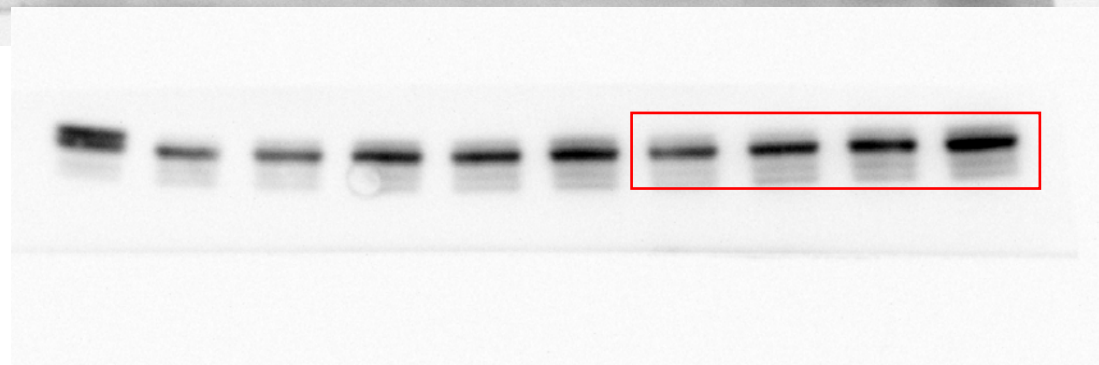

Supplement: Supplementary file 1 [file Data_Sheet_1.PDF]
